# Determinants of Brushite Stone Formation: A Case-Control Study

**DOI:** 10.1371/journal.pone.0078996

**Published:** 2013-11-12

**Authors:** Roswitha Siener, Linda Netzer, Albrecht Hesse

**Affiliations:** University Stone Centre, Department of Urology, University of Bonn, Bonn, Germany; Emory University, United States of America

## Abstract

**Purpose:**

The occurrence of brushite stones has increased during recent years. However, the pathogenic factors driving the development of brushite stones remain unclear.

**Methods:**

Twenty-eight brushite stone formers and 28 age-, sex- and BMI-matched healthy individuals were enrolled in this case-control study. Anthropometric, clinical, 24 h urinary parameters and dietary intake from 7-day weighed food records were assessed.

**Results:**

Pure brushite stones were present in 46% of patients, while calcium oxalate was the major secondary stone component. Urinary pH and oxalate excretion were significantly higher, whereas urinary citrate was lower in patients as compared to healthy controls. Despite lower dietary intake, urinary calcium excretion was significantly higher in brushite stone patients. Binary logistic regression analysis revealed pH>6.50 (OR 7.296; p = 0.035), calcium>6.40 mmol/24 h (OR 25.213; p = 0.001) and citrate excretion <2.600 mmol/24 h (OR 15.352; p = 0.005) as urinary risk factors for brushite stone formation. A total of 56% of patients exhibited distal renal tubular acidosis (dRTA). Urinary pH, calcium and citrate excretion did not significantly differ between patients with or without dRTA.

**Conclusions:**

Hypercalciuria, a diminished citrate excretion and an elevated pH turned out to be the major urinary determinants of brushite stone formation. Interestingly, urinary phosphate was not associated with urolithiasis. The increased urinary oxalate excretion, possibly due to decreased calcium intake, promotes the risk of mixed stone formation with calcium oxalate. Neither dietary factors nor dRTA can account as cause for hypercalciuria, higher urinary pH and diminished citrate excretion. Further research is needed to define the role of dRTA in brushite stone formation and to evaluate the hypothesis of an acquired acidification defect.

## Introduction

The most important calcium phosphates involved in urinary stone disease are dahllite (carbonate apatite) and brushite (calcium hydrogen phosphate dihydrate). Although both minerals contain calcium and phophate, carbonate apatite and brushite are two completely different kinds of stones [1]. Brushite stone disease, albeit rare, has increased in the occurence during the past decades [2], [3], [4]. In a study based on the analysis of more than 50,000 urinary stones, the proportion of brushite-containing stones has been reported to reach 4.3% during the years 2005 to 2009 [3].

Brushite stones are known to grow rapidly with a correspondingly high recurrence rate [5]. Among urinary phosphate stones, brushite has the greatest density and the greatest hardness [6]. Due to their hardness, brushite has been demonstrated to respond poorly to disintegration by extracorporeal shock wave lithotripsy (ESWL) [7], [8]. A study comparing brushite to calcium oxalate stone formers found that brushite stone patients require a greater number of ESWL treatments, not explained by number of stones or duration of stone disease [9]. Brushite stone formers often require multiple surgical interventions to be rendered stone-free [10]. Metabolic evaluation, effective treatment and long-term followup are therefore highly important in brushite stone disease [11].

Unfortunately, there is limited research on the etiology and pathophysiology of brushite stone formation. A low urine volume, a urinary pH in the range of 6.5 to 6.8 and an increased renal excretion of calcium and phosphate are considered to be urinary risk factors for brushite stone formation [12]. Several studies agree that hypercalciuria could represent the most common abnormality in 24-hour urine followed by increased urinary pH [3], [10]. Although possible causes of brushite stone formation include primary hyperparathyroidism [13] and distal renal tubular acidosis (dRTA) [14], Krambeck *et al.* (2010) found no such association in their brushite cohort [10]. Data on underlying metabolic disturbances are sparse, pathogenic mechanisms or etiologic factors are poorly understood and studies on the role of dietary factors in brushite stone disease are lacking. Thus, aim of this case-control study was to identify metabolic abnormalities, dietary and urinary risk factors for brushite stone formation by comparing patients with healthy subjects.

## Subjects and Methods

### Patients

The case group comprised 28 brushite stone formers, 21 men and 7 women, attending our stone clinic with a history of recurrent disease. Exclusion criterion was primary hyperparathyroidism requiring surgery, which is considered to be an apparent predisposing factor for brushite stone formation. The patients were instructed to avoid taking medications that might influence calcium, phosphate, oxalate and purine metabolism or acid-base-status, such as alkaline citrate, allopurinol, thiazides, phosphate binders or vitamin supplements for two weeks before and during investigation. The patients did not receive dietary advice before study participation and were asked to maintain their usual dietary habits during assessment. Stone composition of a recent stone episode was available for all patients in the cohort. The controls were 28 age-, sex- and BMI-matched unrelated healthy individuals. Each subject had a normal physical examination and normal findings from multiparameter urine test strips (Combur9-Test, Boehringer, Mannheim, Germany). No subject had a prior medical history of urolithiasis or other significant diseases. The subjects took no medication or supplementation during evaluation. The profile of patients and controls are shown in Table 1. The study was approved by the ethics committee of the Medical Faculty of the University of Bonn and written informed consent was obtained from all participants.

**Table 1:** Characteristics of study participants.

	Brushite stone formers	Healthy controls	P
	(n = 28)	(n = 28)	
Sex^1^			1.000
Men (n)	21	21	
Women (n)	7	7	
Age (years)^2^	43.5±14.5	43.4±14.6	0.961
Height (cm)^2^	176±9	179±9	0.393
Weight (kg)^2^	82±14	83±15	0.583
BMI (kg/m^2^)^2^	26.1±3.3	25.9±3.6	0.812
Duration of disease (years)^2^	11.6±8.9	–	–

1 Number of patients; Fisher’s exact test.

2 Mean ± SD; Mann-Whitney-U-Test.

### Study Procedure

Anthropometric data, family and patient history, dietary records and 24 h urine samples were collected from participants. Stone formers and controls were studied while consuming their self-selected home diets. Dietary habits of the study participants were assessed by a 7-day weighed food record. The patients provided a detailed description of types and weighed amounts of all food items consumed. The nutrient content of foods was calculated using the computer program PRODI 5.3 (Nutri-Science GmbH, Freiburg, Germany). Oxalate values of foods which have been measured at our laboratory were entered into the software data base [15], [16], [17]. Sodium intake was estimated by the measurement of 24-hour urinary sodium excretion.

### Dietary Oxalate Concentration

For the determination of total oxalate content of foods, oxalate was extracted with 2N hydrochloric acid from homogenized samples. Analysis of filtrates was performed by the HPLC-enzyme-reactor method [18]. Oxalate was separated from matrix substances by an anion exchange column (AS4A-DIONEX, ThermoFisher Scientific, Waltham, Massachusetts). The mobile phase consisted of 2 g/l EDTA solution adjusted to pH 5.0 with 0.3 mol/l NaOH. The enzyme reactor contained 5 units of immobilized oxalate oxidase (oxalate oxidase: Sigma Diagnostics, St. Louis, USA) (carrier: VA Epoxy Biosynth, Riedel-de-Häen, Seelze, Germany) which oxidized oxalate to carbon dioxide and hydrogen peroxide. Resulting hydrogen peroxide was analyzed by amperometric detection.

### 24 h Urinary Excretion Profiles

Urine volume, pH (potentiometry) and concentrations of creatinine (Jaffé reaction), sodium, potassium and chloride (indirect ion selective electrode), calcium (cresolphthalein complex), magnesium (methylthymol blue), inorganic sulfate (nephelometry), inorganic phosphate (phosphate molybdate reaction), ammonium (ion selective electrode), citrate (enzymatically, citrate lyase), uric acid (enzymatically, uricase), and oxalate (enzymatically, oxalate oxidase) were measured. Laboratory quality certification was available for each parameter. The relative supersaturations of calcium oxalate and brushite were calculated using EQUIL2 [19].

### Ammonium Chloride Loading Test

Patients underwent an ammonium chloride loading test to identify dRTA [11]. The ammonium chloride loading test consisted of sampling fasting venous blood and fractional 24 h urine under standardized conditions. The amount of administered ammonium chloride depended on the body weight of patients (0.1 g NH_4_Cl per kg body weight). According to the urinary pH in the day profile (persistent urinary pH above 5.4) and results of blood analysis, complete and incomplete dRTA could be distinguished.

### Stone Analysis

Infrared spectroscopy was used for stone analysis. Patients had at least one stone containing a minimum of 40% of brushite. The percentage of brushite and secondary components were recorded. Laboratory quality certification was available for stone analysis.

### Statistics

Comparisons between groups were performed using the Mann-Whitney U-test for non-parametric unpaired data. Categorical variables were compared with Fisheŕs exact test. Receiver operating characteristic analysis was performed to determine the AUC, sensitivity and specificity of risk factors. Binary logistic regression was used to denote the extent of risk interference. Data are presented as means ± standard deviation. All reported P values are two-sided. Differences were considered significant at p<0.05. Statistical analysis was performed using IBM Statistical Package for the Social Sciences version 21.0 (SPSS Inc., Chicago, IL, USA).

## Results

### Characteristics

The characteristics of patients and healthy controls are shown in Table 1. The case group comprised 28 patients with brushite stones, 21 (75%) men and 7 (25%) women. The control group consisted of 28 unrelated healthy subjects without a history of urolithiasis.

Stone composition was available for all patients in the cohort. Pure brushite stones were present in 13 out of 28 (46%) patients. Brushite mixed with calcium oxalate was noted in 9 (32%), mixed with carbonate apatite in 3 (11%), calcium oxalate plus carbonate apatite in 2 (7%) and calcium oxalate plus struvite in 1 (4%) patient. In patients with mixed stones, the percentage of brushite was 72±18% (n = 15) and of calcium oxalate 25±16% (n = 12).

A family history of stones was found in 11 out of 27 patients (41%), among which at least one parent was involved in 9 out of 27 patients (33%). Out of 25 patients for whom ammonium chloride loading test had been performed 14 patients (56%), i.e. 53% (10/19) of men and 67% (4/6) of women (p = 0.661), had incomplete dRTA.

### Dietary Intake

Dietary intake of calcium was significantly lower in brushite stone formers compared to controls (744±271 vs. 992±466 mg/d; p = 0.027) (Table 2). Other dietary factors did not differ between groups.

**Table 2:** Dietary intake of brushite stone formers and healthy controls (mean ± standard deviation).

	Brushite stone formers	Healthy controls	P
	(n = 28)	(n = 28)	
Energy (kcal/d)	2,336±533	2,531±516	0.232
Energy (kJ/d)	9,744±2,249	10,516±2,222	0.302
Protein (g/d)	85±19	93±22	0.251
Protein (g/kg body weight/d)	1.06±0.26	1.14±0.26	0.334
Carbohydrates (g/d)	267±65	277±75	0.743
Fat (g/d)	88±23	96±25	0.486
Cholesterol (mg/d)	330±103	334±137	0.502
Dietary fiber (g/d)	21.5±6.0	21.6±8.0	0.915
Sodium (g/d)	4.3±1.3	5.1±2.1	0.184
Potassium (mg/d)	3,130±805	3,019±673	1.000
Magnesium (mg/d)	356±99	406±153	0.222
Calcium (mg/d)	744±271	992±466	0.027
Total oxalate (mg/d)	112±48	93±46	0.091
Soluble oxalate (mg/d)	57±25	50±26	0.190
Purines (mg/d)	368±104	348±144	0.258
Phosphorus (mg/d)	1,198±320	1,264±302	0.422
Ascorbic acid (mg/d)	127±73	105±56	0.481
Thiamine (mg/d)	1.41±0.58	1.22±0.35	0.235
Pyridoxine (mg/d)	2.01±0.93	1.83±0.58	0.561
Alcohol (g/d)	13.9±16.5	22.9±22.0	0.057
Total fluid (ml/d)	3,462±778	3,475±842	0.935

### Urinary Parameters

Urinary pH was higher (p = 0.002) and citrate excretion lower (p<0.001) in patients compared with healthy controls (Table 3). Despite lower dietary calcium intake (p = 0.027) urinary calcium excretion was higher (p<0.001) in brushite stone patients. Urinary oxalate was higher (p = 0.027), phosphate similar (p = 0.232) and chloride excretion lower (p = 0.047) in patients than controls. The relative supersaturation of brushite and calcium oxalate were two times higher (all p<0.001) among patients than controls. Other urinary parameters did not differ between groups. Hypercalciuria, defined as urinary calcium excretion exceeding 5 mmol/24 h in both sexes [11], was found in 27 out of 28 (96%) patients and in 11 out of 28 (39%) controls (p<0.001).

**Table 3:** Urinary parameters in brushite stone formers and healthy controls (mean ± standard deviation).

	Brushite stone formers	Healthy controls	P
	(n = 28)	(n = 28)	
Volume (l/24 h)	2.519±0.930	2.558±1.017	0.967
pH	6.57±0.34	6.18±0.48	0.002
Density (g/cm^3^)	1.009±0.005	1.012±0.006	0.119
Sodium (mmol/24 h)	186±56	220±93	0.184
Potassium (mmol/24 h)	72±25	85±26	0.059
Calcium (mmol/24 h)	8.41±2.38	4.96±2.55	<0.001
Magnesium (mmol/24 h)	5.44±2.15	5.41±2.28	0.838
Ammonium (mmol/24 h)	25.7±11.0	21.2±7.2	0.132
Chlorid (mmol/24 h)	178±68	226±97	0.047
Phosphate (mmol/24 h)	34.7±10.0	39.7±14.0	0.232
Sulphate (mmol/24 h)	22.6±6.1	27.7±11.8	0.120
Creatinine (mmol/24 h)	14.98±3.61	15.94±4.34	0.403
Urate (mmol/24 h)	3.88±1.04	4.70±1.64	0.054
Oxalate (mmol/24 h)	0.433±0.102	0.363±0.126	0.027
Citrate (mmol/24 h)	2.405±0.967	3.943±1.321	<0.001
RS Brushite	2.15±0.87	0.82±0.77	<0.001
RS CaOx	6.80±3.07	3.22±2.25	<0.001

RS: relative supersaturation.

**Figure 1:**
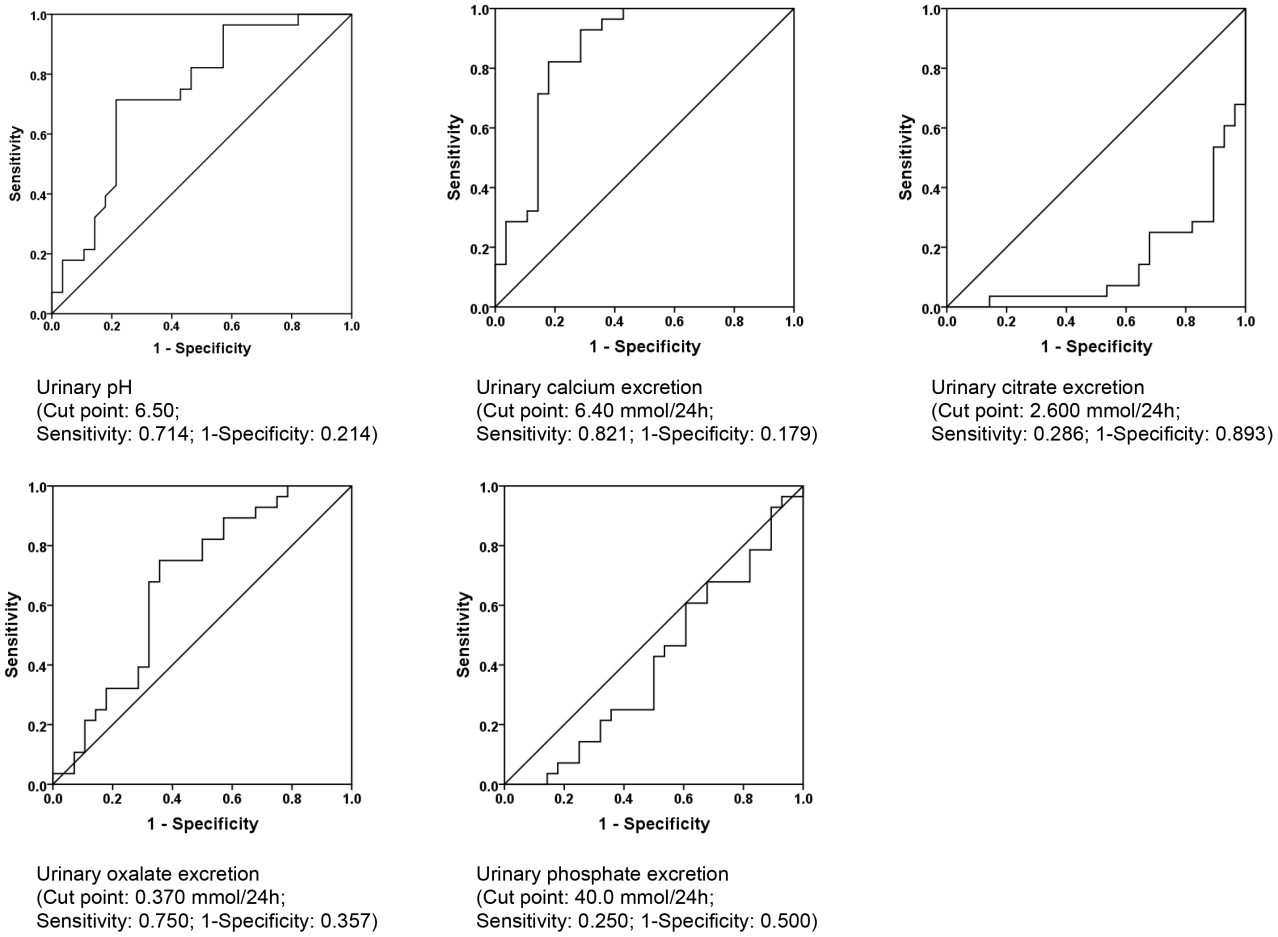
Receiver operating characteristic analyses.

Receiver operator characteristic analysis identified cut-points associated with urolithiasis (Figure 1). Binary logistic regression analysis revealed pH >6.50 (OR 7.296; p = 0.035), calcium >6.40 mmol/24 h (OR 25.213; p = 0.001) and citrate excretion <2.600 mmol/24 h (OR 15.352; p = 0.005) as major urinary risk factors for brushite stone formation (Table 4). An increase in urinary calcium excretion by 0.01 mmol/24 h increased the risk of stone formation by 0.8% (p = 0.001), whereas an inverse relation was found for urinary citrate excretion. Interestingly, urinary phosphate excretion was not associated with brushite stone formation.

**Table 4:** Binary logistic regression.

	Cut point	Odds ratio forcut point	95% Wald confidence limits	p	Odds ratio for increaseby 0.01 mmol/24 h	p
Urinary pH	>6.50	7.296	1.146; 46.433	0.035	–	–
Urinary calcium (mmol/24 h)	>6.40	25.213	3.646; 174.326	0.001	1.008	0.001
Urinary citrate (mmol/24 h)	<2.600	15.352	2.238; 105.292	0.005	0.980	0.001
Urinary oxalate (mmol/24 h)	>0.370	–	–	–	–	–
Urinary phosphate (mmol/24 h)	<40.0	–	–	–	–	–

Urinary pH (6.71±0.23 vs. 6.45±0.40; p = 0.189), calcium (7.75±2.04 vs. 9.20±2.42; p = 0.071) and citrate excretion (2.397±1.086 vs 2.515±0.756; p = 0.511) did not statistically significant differ between patients with (n = 14) or without dRTA (n = 11).

## Discussion

Brushite-containing stones are highly recurrent, particularly hard and physically resistant to ESWL [5], [6], [7], [8]. Brushite stone formers therefore require aggressive intervention, comprehensive metabolic evaluation and long-term follow-up for stone disease. So far, the pathogenic factors driving the development of brushite stones are not known. We found that men predominate among brushite stone formers. Our results confirm the observations of Krambeck *et al.* (2010) [10] and Parks *et al.* (2004) [9] that more men than women were affected by brushite stone formation, which corresponds to the gender distribution in calcium oxalate stone disease. Interestingly, calcium oxalate was the major secondary stone component in our cohort.

Distal RTA has been reported to occur in 3% of calcium oxalate stone formers and in 32% of patients with brushite stones [14]. According to our findings, 56% of the brushite stone patients exhibited incomplete dRTA, suggesting that dRTA could be even more common in brushite stone formers than previously noted. Brushite stone formation in dRTA may result from hypercalciuria, low urinary citrate and high urinary pH, which will increase brushite crystallization [20]. Although dRTA is considered a major risk factor for brushite stone formation, studies on the effect of dRTA on urinary risk profile of brushite stone formers are lacking. In the current study, urinary pH, calcium and citrate excretion did not significantly differ between patients with and without dRTA.

Hypercalciuria has been reported to be the most common abnormality in 24-hour urine of brushite stone patients [3], [10]. Our case-control study confirmed that hypercalciuria is a key determinant of brushite stone formation. Besides dRTA, several pathogenetic mechanisms leading to hypercalciuria, including dietary factors, increased intestinal absorption and reduced renal tubular reabsorption of calcium (“renal leak”), should be considered [21], [22]. Because urinary calcium excretion did not differ between patients with or without dRTA, it is unlikely that dRTA might be the major cause of hypercalciuria in our cohort. Moreover, dietary factors, particularly high intake of calcium, sodium and protein may contribute to increased urinary calcium excretion [23], [24]. Whereas dietary calcium intake was significantly lower in patients than controls, dietary sodium and protein intake was similar in both groups. Importantly, the mean dietary intake of calcium in brushite stone formers was 744 mg/d, which is far below the dietary recommendation of at least 1000 mg calcium/d [25]. Thus, the cause for the higher calcium excretion in our cohort of patients remains unclear.

Higher urinary pH favors calcium phosphate crystallization [5], [9]. Whereas carbonate apatite occurs at urinary pH values greater than 6.9, brushite is more likely to form in weakly acidic urine with a pH in the range of 6.5 and 6.8 and may transform to carbonate apatite at pH values of 6.9 and above [5], [14]. In the current study, urinary pH was significantly higher in patients compared to controls. Because neither dRTA nor dietary factors can account as cause for higher urinary pH in our cohort of patients, the mechanism of increased urinary pH in brushite stone formers is unknown. Interestingly, urinary phosphate excretion did not differ between patients and healthy controls. This study suggests that calcium, but not phosphate is an important determinant of brushite stone formation and that the persistently elevated urinary pH highly favors the precipitation of brushite. Moreover, the significantly lower urinary citrate excretion in patients compared to controls is a pathogenetically important risk factor for brushite stone formation. In the absence of dietary risk factors, the lower urinary citrate excretion observed in our patients is suggested to result from acid retention inducing intracellular acidosis in proximal tubular cells, which favors reabsorption of citrate [26].

To our knowledge, studies examining the role of dietary intake and urinary excretion of oxalate in brushite stone formation are lacking. In the present study, urinary oxalate excretion was higher in brushite stone patients than in healthy controls, whereas dietary oxalate intake did not significantly differ between both groups. Our results are in accordance with findings of a retrospective study conducted by Parks *et al.* (2009), who noted a significantly higher urinary oxalate excretion in patients in whom transformation from calcium oxalate to calcium phophate stones occurred over time than in calcium oxalate controls who did not transform [27]. However, a major limitation of their study is that no distinction was made between patients with brushite stones and those with stones of carbonate apatite, which is another phase of calcium phosphate. Increased urinary oxalate excretion, possibly due to decreased calcium intake, promotes the risk of mixed stone formation with calcium oxalate. Our study revealed that the relative supersaturation of brushite but also of calcium oxalate was significantly higher in patients with brushite stones than in healthy controls, largely due to higher urinary calcium and oxalate and lower citrate excretion. Because calcium oxalate was the major secondary component of brushite stones, evidence exists to support the hypothesis that conversion from calcium oxalate to brushite stone disease could have occured over time [27]. It has been assumed that pH dysregulation due to renal injury from obstruction or other causes, such as ESWL or alkalinization therapy, is the initiating factor for this conversion [28]. Further research assessing the role of crystal-induced nephron damage in brushite stone formation is required to evaluate the hypothesis of an acquired acidification defect.

## Conclusions

The present study emphasizes that urinary calcium and citrate, but not phosphate excretion are important determinants of brushite stone formation and that the elevated urinary pH highly favors the precipitation of brushite. The increased urinary oxalate excretion, possibly due to decreased calcium intake, promotes the risk of mixed stone formation with calcium oxalate. Neither dietary factors nor dRTA can account as cause for hypercalciuria, higher urinary pH and diminished citrate excretion in our cohort of patients. Further research is needed to define the role of dRTA in brushite stone formation and to assess the hypothesis of an acquired acidification defect.
